# Effects of human chorionic gonadotropin on echotextural attributes of the original corpus luteum in Morada Nova ewes

**DOI:** 10.1007/s11250-026-04998-8

**Published:** 2026-03-24

**Authors:** Joedson Dantas Gonçalves, Gabriel Brun Vergani, Juliana Nascimento Duarte Rodrigues, Jenniffer Hauschildt Dias, Verônica Schinaider do Amaral Pereira, Alexandre Rossetto Garcia, Sérgio Novita Esteves, Jeferson Ferreira da Fonseca, Maria Emilia Franco Oliveira

**Affiliations:** 1 School of Agricultural and Veterinary Sciences, São Paulo State University (UNESP), Via de Acesso Prof. Paulo Donato Castellane, s/n, Jaboticabal, SP 14884-900 Brazil; 2 Federal Rural University of the Amazon (UFRA), Paragominas Campus, PA- 256 Highway, s/n, Paragominas, PA 68627-451 Brazil; 3 Federal University of Viçosa (UFV), Peter Henry Rolfs Avenue, s/n, Viçosa, MG 36570-900 Brazil; 4 Embrapa Southeast Livestock, Rodovia Washington Luiz, km 234, São Carlos, SP 13560-970 Brazil; 5 Embrapa Goats and Sheep, Estrada Sobral/Groaíras, km 04, P.O. Box 145, Sobral, CE 62010-970 Brazil; 6 Faculty of Veterinary Medicine, Autonomous University of Barcelona, Bellaterra, 08193 Spain

**Keywords:** Luteal tissue, Echotexture, Doppler ultrasonography, Luteotropic effect, Sheep

## Abstract

This study evaluated whether human chorionic gonadotropin (hCG) affects biometric, echotextural, and signs color Doppler variables of the original corpus luteum (oCL) in Morada Nova ewes. Sixty-four ewes underwent estrus synchronization with intravaginal sponges impregnated with 60 mg of medroxyprogesterone acetate for six days, with an intramuscular injection of 37.5 µg of d-cloprostenol and 200 IU of equine chorionic gonadotropin thirty-six hours before sponge removal. The ewes were subjected to natural mating and subsequently allocated to receive 300 IU of hCG (hCG group; *n* = 34) or 1 mL of saline solution (Control group; *n* = 30) intramuscularly 7.5 days after sponge removal. Biometric variables of the corpus luteum (diameter, area, volume), echotextural variables (numerical pixels values ​​and heterogeneity), and color Doppler signals (color Doppler area, color Doppler volume, and percentage of perfusion) were evaluated on days 7.5, 10.5, 13.5, and 21.5 after sponge removal. The oCL showed no differences in biometric or color Doppler signals between the hCG and control groups (*P* > 0.05). However, hCG increased the echotextural variables, with oCL exhibiting higher numerical pixel values ​​(88.8 ± 4.7^a^ vs. 70.0 ± 0.8^b^; *P* = 0.0001) and heterogeneity (14.3 ± 0.6^a^ vs. 11.5 ± 0.2^b^ 38; *P <* 0.0001) compared to the control 39 oCL, respectively. Furthermore, this increased echotextural variables was also observed in pregnant females with oCL hCG when compared to pregnant females with oCL control (*P* < 0.05). These findings indicate that hCG enhances echotextural attributes of the oCL, suggesting functional improvement rather than structural or vascular changes during the early luteal phase.

## Introduction

The use of gonadotropins with luteotrophic effects has been extensively studied as a strategy to improve conception rates in ewes and goats (Fonseca et al. 2018; Vergani et al. 2020; Côrtes et al. 2021; Rodrigues et al. 2023a, b, 2025; Martins et al. 2025). The intramuscular administration of human chorionic gonadotropin (hCG) has been widely investigated due to its luteotropic effects in small ruminants. Previous studies have demonstrated its role in preventing early corpus luteum regression (Farin et al. 1988), as well as increasing serum progesterone (P4) concentrations in goats (Rodrigues et al. 2022, 2025) and ewes (Fonseca et al. 2018; Vergani et al. 2020). Furthermore, through its luteotropic effect, hCG promotes an increase in the area or volume of luteal tissue (Fonseca et al. 2018; Vergani et al. 2020; Rodrigues et al. 2022, 2023a, b, 2025) and enhances ovarian blood perfusion (Vergani et al. 2020; Dias et al. 2022; Rodrigues et al. 2023b, 2025). Consequently, improvements in pregnancy rates have also been reported, particularly in goats (Côrtes et al. 2021; Rodrigues et al. 2022, 2025) and, to a lesser extent, in ewes (Nephew et al. 1994; Khan et al. 2003; Fonseca et al. 2021).

The ability of hCG to induce accessory corpora lutea (aCL) is well-documented in sheep (Coleson et al. 2015; Fonseca et al. 2018, 2021; Vergani et al. 2020; Gonçalves et al. 2024) and goats (Côrtes et al. 2021; Fonseca et al. 2021; Rodrigues et al. 2022, 2025). However, it has long been suggested that the effect of hCG is not limited to aCL formation (Schmitt et al. 1996). This is evidenced by increased circulating P4 concentrations in heifers that did not develop aCL following hCG administration five days after estrus. Similarly, buffaloes with both an original corpus luteum (oCL) and an aCL exhibited plasma P4 concentrations comparable to those with only an oCL (Pandey et al. 2015). The distinct effects of hCG on oCL and aCL can now be assessed by ultrasonography, although studies remain limited. However, ultrasound techniques, specifically echotextural analyses, can be used to evaluate luteal tissue function, as they are non-invasive snd easy perfom. The numerical pixel values ​​and heterogeneity directly reflect differences in tissue density and tissue composition (Arashiro et al. 2010; Bevilaqua et al. 2023). A higher luteal tissue density may be directly related to a greater proportion of steroidogenic cells, consequently leading to increased progesterone production and more efficient luteal function (Duggavathi et al. 2003).

In cattle, (Rizos et al. 2012) reported an increase in oCL area after administering 3000 IU of hCG on day 5 post-estrus, with similar results observed in buffaloes (Pandey et al. 2015). In goats, recent studies have demonstrated that 300 IU of hCG administered seven days after synchronized estrus significantly increased plasma P4 concentrations and pregnancy rates, these improvements were associated with both the induction of aCL and a hypertrophic effect on the existing oCL (Rodrigues et al. 2022, 2025). Reinforcing these narratives, Fernández et al. (2024) demonstrated that ewes treated with hCG, regardless of whether or not aCL was formed, showed high expressions of steroidogenic enzymes, such as acute regulatory protein transport (STAR) and enzyme 3β hydroxysteroid dehydrogenase (HSD3B1), which confirms the luteotropic effect of this gonadotropin on the oCL. However, there are still no studies evaluating this luteotropic effect specifically on the ultrasonographic variables of the oCL in isolation in sheep, whether treated with hCG or not.

Based on previous findings, we hypothesize that hCG exerts a luteotropic effect not only through the induction of aCL but also by directly stimulating the oCL in ewes. However, this effect on the oCL may be less pronounced when aCL formation occurs. Therefore, the objective of the present study was to evaluate the effects of administering 300 IU of hCG 7.5 days after progestin sponge removal on the biometric (diameter, area, and volume), echotextural (numerical pixels values and pixel heterogeneity), and color Doppler (vascular area, volume, and percentage relative to total luteal area) variables of oCL and aCL, assessed separately, in Morada Nova ewes during the breeding season.

## Materials and methods

### Location, animals and management

The experiment was carried out at the Canchim Farm, Embrapa Southeast Livestock, located in São Carlos, SP, Brazil (21°57′28.5″ S and 47°50′36.6″ W), during the breeding season (April to May), under natural photoperiod, with an average temperature of 22 °C and relative humidity of approximately 65%.

Sixty-four Morada Nova ewes, clinically healthy, multiparous, with an average age of 3.5 ± 1.2 years, body weight (BW) of 40.6 ± 0.6 kg, and body condition score (BCS) of 3.3 ± 0.1 (on a scale 1–5), were reared under a semi-intensive system, with rotational grazing on *Panicum maximum* cv. Aruana pastures and free access to water and mineral salt. The diet was supplemented with consisted of corn silage and 200 g/animal/day of a concentrate containing 16% crude protein and 72% total digestible nutrients. Prior to the synchronization protocol, cyclic ovarian activity was confirmed in all animals via transrectal ultrasonography.

### Estrus synchronization, mating and treatments

**Fig. 1:**
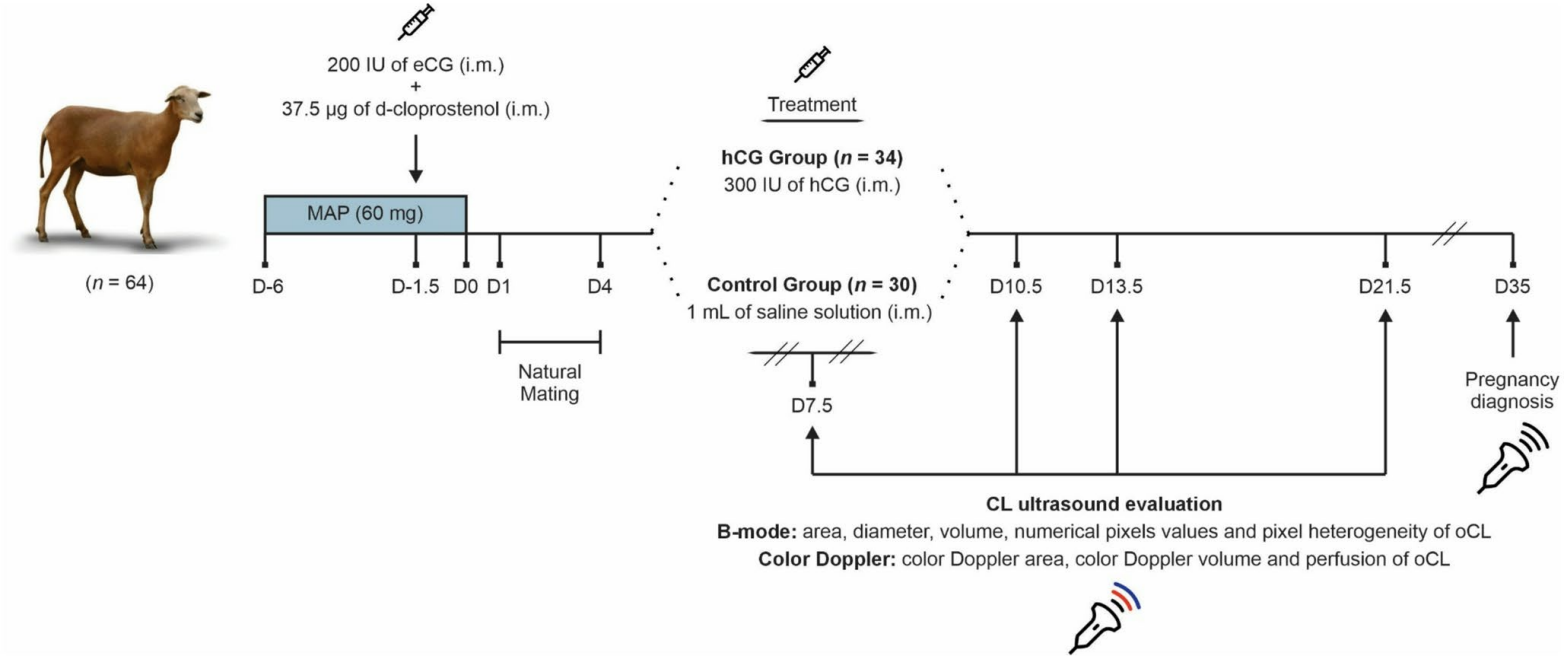
Experimental study design to analyzed the effects of 300 IU of hCG (i.m.) administered on day 7.5 after sponge removal on original corpus luteum (oCL) and accessory corpora lutea (aCL) variables. Synchronization protocol - (MAP; Progespon^®^, Zoetis, USA) for six days, 36 h before sponge removal, 37.5 µg of d-cloprostenol (i.m. Veteglan^®^, Hertape Calier, Spain) and 200 IU of eCG (i.m. Novormon^®^, Zoetis, USA). Natural mating was performed from day 1 to day 4 after sponge removal. On day 7.5, 300 IU of hCG (i.m.) or 1 mL saline solution was administrated. B-mode and color Doppler variables of luteal structures were performed on days 7.5, 10.5, 13.5 and 21.5. The pregnancy diagnosis was performed on D35

All ewes received intravaginal sponges impregnated with 60 mg of medroxyprogesterone acetate (MAP; Progespon^®^, Zoetis, USA), which were inserted in the late afternoon (between 5:00 and 6:00 p.m.) and remained in place for six days. Thirty-six hours before sponge removal (Day − 1), an intramuscular (i.m.) injection of 37.5 µg of d-cloprostenol (Veteglan^®^, Hertape Calier, Spain) and 200 IU of equine chorionic gonadotropin (eCG; Novormon 5000^®^, Zoetis, Brazil) was administered. After sponge removal (Day 0), the ewes were naturally mated with four fertile rams (at a ram: ewe ratio of 1:16) for three consecutive days. Rams were previously evaluated and classified as reproductively sound according to the Brazilian College of Animal Reproduction (CBRA 2013). The experimental design is illustrated in Fig. 1.

On day 7.5 (between 7:00 and 8:00 a.m.) following sponge removal, the females were equally divided into two experimental groups according to BW, BCS, and the ram used for mating. The sheep were designated to receive an im. injection of 300 IU of hCG (Vetecor^®^, Hertape-Calier, Spain) (G-hCG, *n* = 34; BW: 40.7 ± 0.6 kg, and BCS: 3.3 ± 0.1) or 1 mL of saline solution (NaCl 0.9%, Eurofarma Lab SA, Brazil) (G-Control, *n* = 30; BW: 40.6 ± 0.7 kg, and BCS: 3.4 ± 0.1). The selected dose of 300 IU of hCG was based on previous studies from our research group, which demonstrated its luteotropic effect, as well as its ability to induce aCL formation and increase progesterone secretion in small ruminants (Fonseca et al. 2018; Vergani et al. 2020; Côrtes et al. 2021; Rodrigues et al. 2022).

### Luteal evaluation and pregnancy diagnosis

Transrectal ovarian ultrasonography was performed one week before the estrus induction protocol, to assess reproductive seasonality and on days 7.5, 10.5, 13.5, and 21.5 of the synchronized estrous cycle (day 0 = sponge removal). Evaluations were conduceted using a MyLab30 VET Gold^®^ ultrasound device (Esaote, Genova, Italy) equipped with a multifrequency linear transrectal transducer (6–8 MHz). The transducer was attached to a polyvinyl chloride (PVC) tube to facilitate external manipulation during the transrectal examination. The ewes were kept in a quadrupedal position during the examination. Feces were manually removed from the rectal ampoule, and 10 mL of carboxymethylcellulose gel (Carbogel UTL; Carbogel Industry and commerce LTDA, São Paulo, SP, Brazil) was deposited with a syringe into the rectum and approximately 5 mL was applied to the transducer before each examination. All evaluations were performed under standardized settings (pulse repetition frequency (PRF): 1.4 kHz; color gain: 64%; depth: 8 cm; Doppler frequency: 5 MHz; and a wall filter: 75 Hz) and with an insonation angle ≤ 30° to ensure consistency in color Doppler measurements.

For the biometric analysis, the B-mode images were analyzed using ImageJ^®^ software (National Institutes of Health, Bethesda, MD, USA) to mensure the diameter (mm), area (mm²), and volume (mm³) of the oCL and aCL. Evaluations were performed on the largest cross-section of each detected CL. If more than one CL was observed, the sum was calculated. The estimated volume of the CL was calculated using the formula for the volume of a sphere (4/3 × π × r³), with r = radius and π = 3.1416. For cavitary CL, the dimensions of the cavity were subtracted. Furthermore, the images were carefully analyzed to identify ewes with oCL without aCL formation and ewes with oCL with aCL present in the contralateral and ipsilateral ovary. Differentiation between the oCL and the aCL in the ipsilateral ovary was only possible on day 13.5, as the aCL were still visibly smaller. By day 21.5, it was no longer possible to differentiate the oCL from the aCL in the ipsilateral ovary.

Echotextural analysis was performed using B-mode images and processed in Image Pro Plus 7.0™ software (Media Cybernetics Inc., USA), where numerical grayscale pixel values ​​from 0 (absolute black) to 255 (absolute white). The values ​​were obtained from a circle formed in the largest possible area of ​​each CL, thus generating the mean numerical pixels values ​​(NPV) and heterogeneity (standard deviation of NPV). In cavitary CL, the cavity was not included in the analysis.

The analysis of color Doppler signals was initially performed using Adobe Fireworks^®^ software (Adobe Systems Incorporated, San Jose, CA, USA). For each evaluation, the image of the isolated CL with the largest area of ​​visualized perfusion was extracted. Subsequently, the image was submitted to ImageJ^®^ software (National Institutes of Health, Bethesda, MD, USA) for pixel counting. The color Doppler area (mm²) was calculated based on the number of colored pixels, and color Doppler volume (mm³) was estimated using the formula: (average percentage of perfusion × summed luteal tissue volume) ÷ 100. The perfusion was calculated as: (colored pixels ÷ total luteal pixels) × 100 (Vergani et al. 2020).

All image analyses were performed by the same evaluator blinded to treatment allocation, and each measurement was repeated three times to calculate an average value.

Pregnancy was diagnosed 35 days after mating using the same ultrasound equipment. The presence of an embryonic vesicle and a fetal heartbeat was used to confirm pregnancy. Subsequently, pregnancy diagnosis (PD) was used as a fixed effect precisely because, after maternal-fetal recognition or its absence, hormonal and structural changes occur in the female reproductive system, leading to the onset of luteolysis (Figueira et al. 2015). These alterations could affect the results if differentiation between pregnant and non-pregnant females were not used, precisely because it can mask these differences in volume, echotextural variables, and perfusion of the CL.

### Statistical analyses

Data were tested for normality using the Shapiro–Wilk test and transformed using the Box-Cox method when necessary (Box and Cox 1964). Statistical analyses were performed using the PROC MIXED (Littell et al. 1996) procedure with repeated measures in SAS (version 9.4; SAS Institute Inc., Cary, USA), considering treatment (Control vs. hCG), day (7.5, 10.5, 13.5, and 21.5), pregnancy diagnosis (Pregnant vs. Non-pregnant), and their interactions as fixed effects. For the analysis of the average number of CL per animal, the Student’s t-test was performed. For comparisons on day 21.5 involving PD, only pregnant animals were included, and means were compared using the Student’s t-test. Results are presented as least squares means ± standard error of the mean (SEM). Differences were considered significant at *P* ≤ 0.05.

**Fig. 2:**
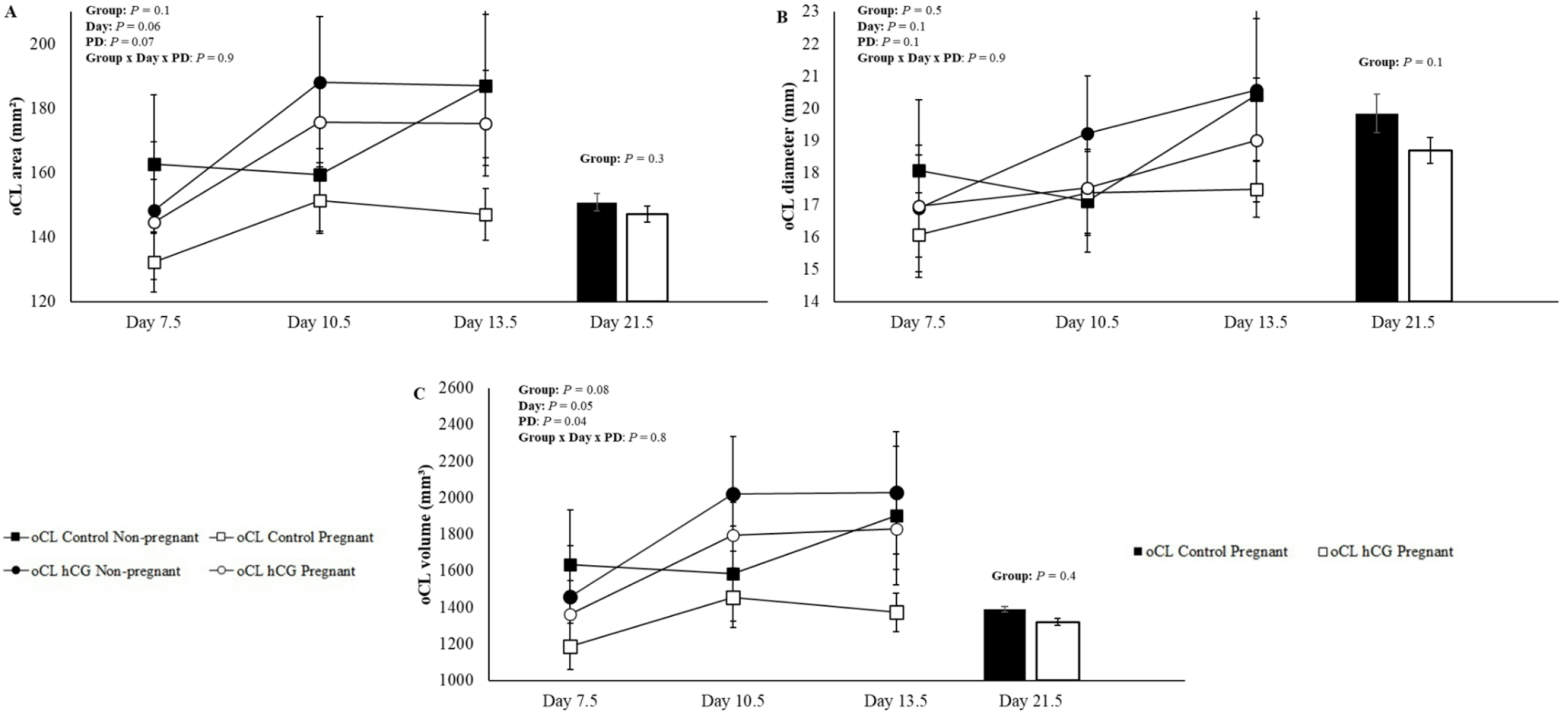
Mean (± SEM) area (**A**), diameter (**B**), volume (**C**) of the original corpus luteum (oCL) on days 7.5, 10.5, 13.5, and 21.5 of Morada Nova ewes pregnant and not pregnant that received i.m. administration of 300 IU of hCG (G-hCG) or 1 mL of saline (G-Control) on Day 7.5 after synchronized estrus induction treatment in the breeding season; *Different superscript letters indicate differences (*P* ≤ 0.05). **PD = Pregnancy diagnosis. *Group *=* G-hCG (*n* = 25) and G-Control (*n* = 30). *oCL Control Non-pregnant (*n* = 9); oCL Control Pregnant (*n* = 21); oCL hCG Non-pregnant (*n* = 11) and oCL hCG Pregnant (*n* = 14). *For G-hCG (*n* = 25), data were used from sheep with oCL hCG without aCL formation and sheep with oCL with aCL present in the contralateral ovary

**Fig. 3:**
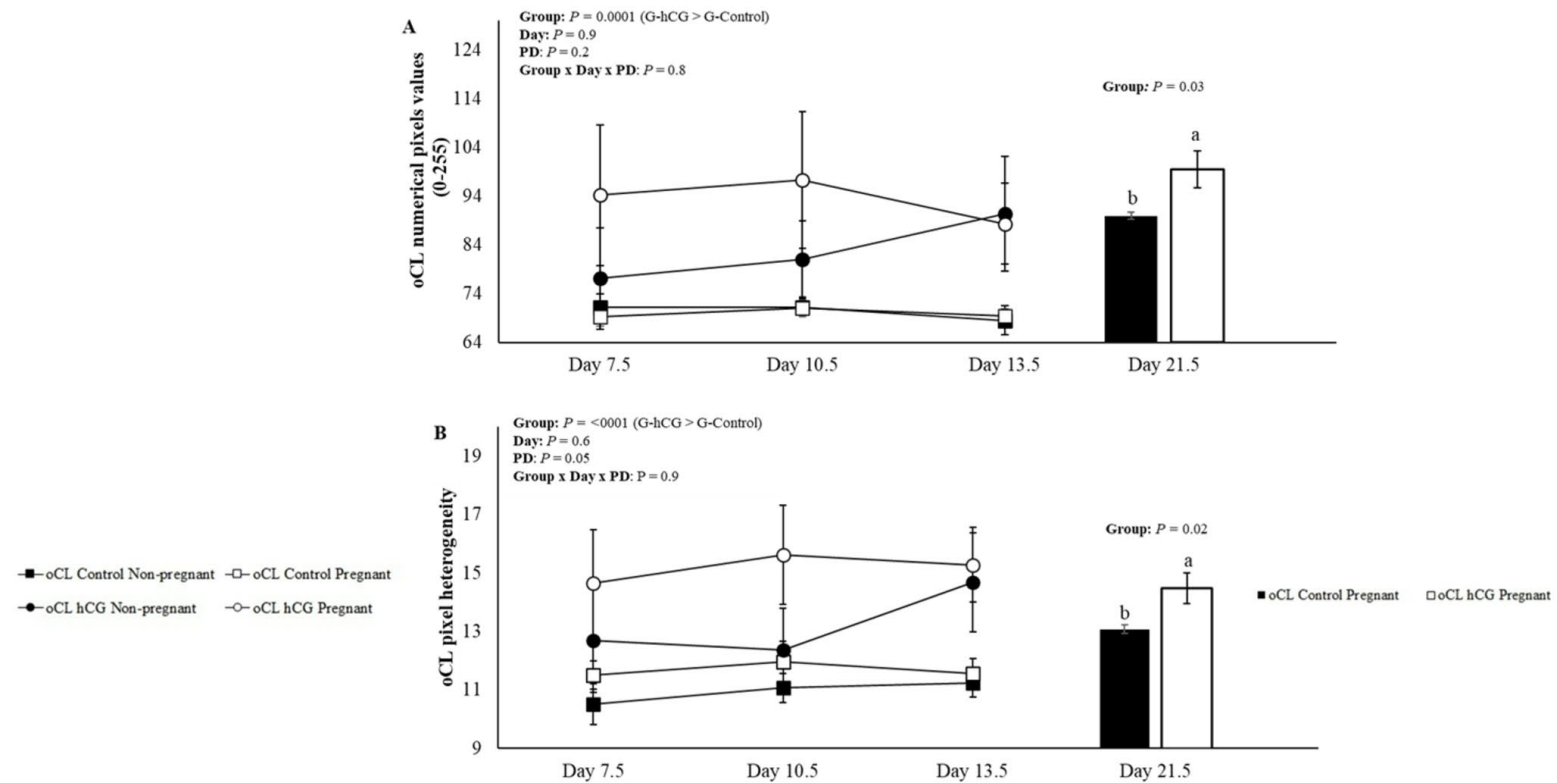
Mean (± SEM) of numerical pixels values (**A**) and pixel heterogeneity (**B**) of the original corpus luteum (oCL) on days 7.5, 10.5, 13.5, and 21.5 of Morada Nova ewes pregnant and not pregnant that received i.m. administration of 300 IU of hCG (G-hCG) or 1 mL of saline (G-Control) on Day 7.5 after synchronized estrus induction treatment in the breeding season; *Different superscript letters indicate differences (*P* ≤ 0.05). **PD = Pregnancy diagnosis. *Group *=* G-hCG (*n* = 25) and G-Control (*n* = 30). *oCL Control Non-pregnant (*n* = 9); oCL Control Pregnant (*n* = 21); oCL hCG Non-pregnant (*n* = 11) and oCL hCG Pregnant (*n* = 14). *For G-hCG (*n* = 25), data were used from sheep with oCL hCG without aCL formation and sheep with oCL with aCL present in the contralateral ovary

**Fig. 4:**
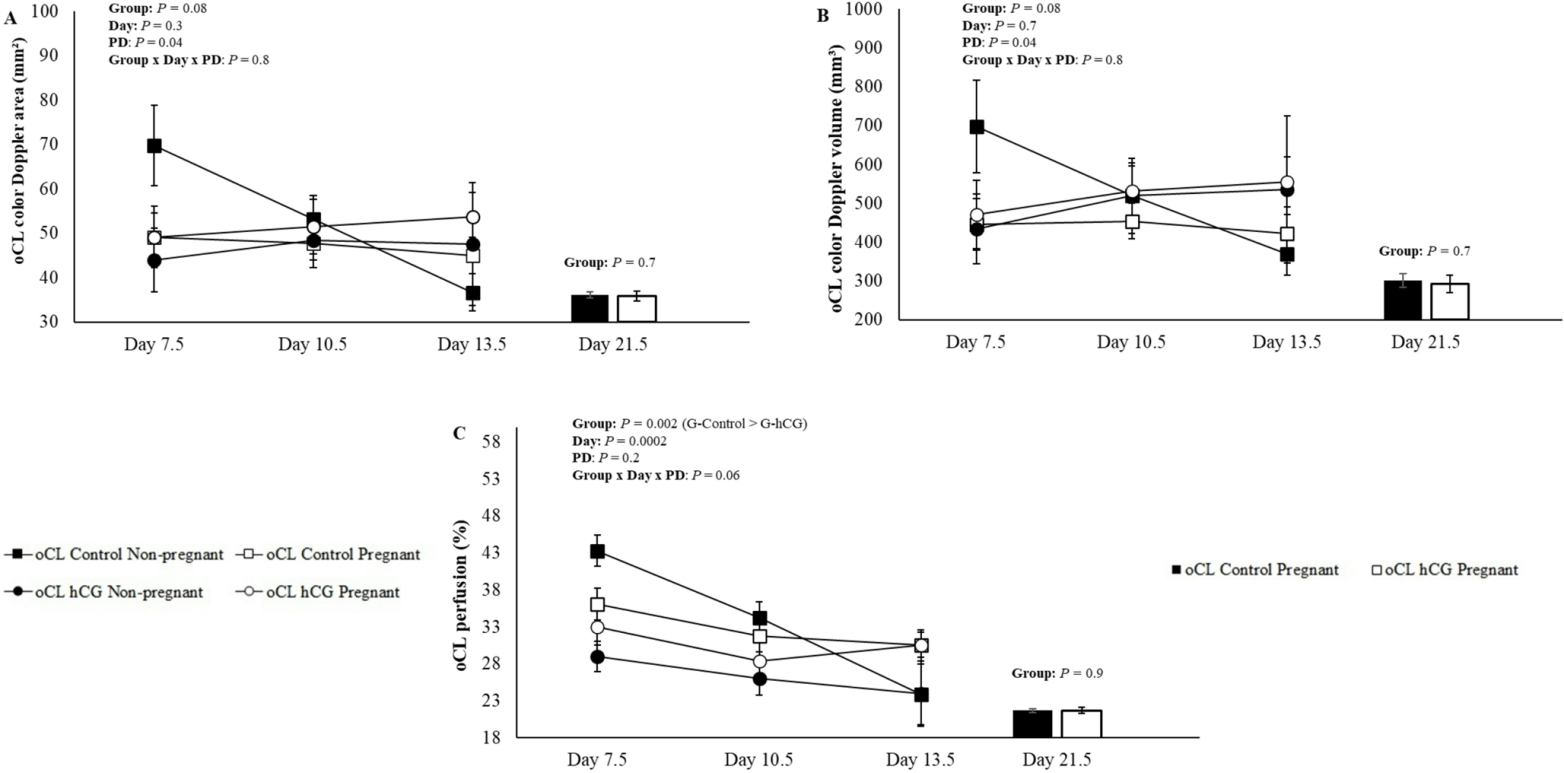
Mean (± SEM) color Doppler area (**A**), color Doppler volume (**B**) and perfusion (**C**) of the original corpus luteum (oCL) on days 7.5, 10.5, 13.5 and 21.5 of Morada Nova ewes pregnant and not pregnant that received i.m. administration of 300 IU of hCG (G-hCG) or 1 mL of saline (G-Control) on Day 7.5 after synchronized estrus induction treatment in the breeding season; *Different superscript letters indicate differences (*P* ≤ 0.05). **PD = Pregnancy diagnosis. *Group *=* G-hCG (*n* = 25) and G-Control (*n* = 30). *oCL Control Non-pregnant (*n* = 9); oCL Control Pregnant (*n* = 21); oCL hCG Non-pregnant (*n* = 11) and oCL hCG Pregnant (*n* = 14). *For G-hCG (*n* = 25), data were used from sheep with oCL hCG without aCL formation and sheep with oCL with aCL present in the contralateral ovary

**Fig. 5:**
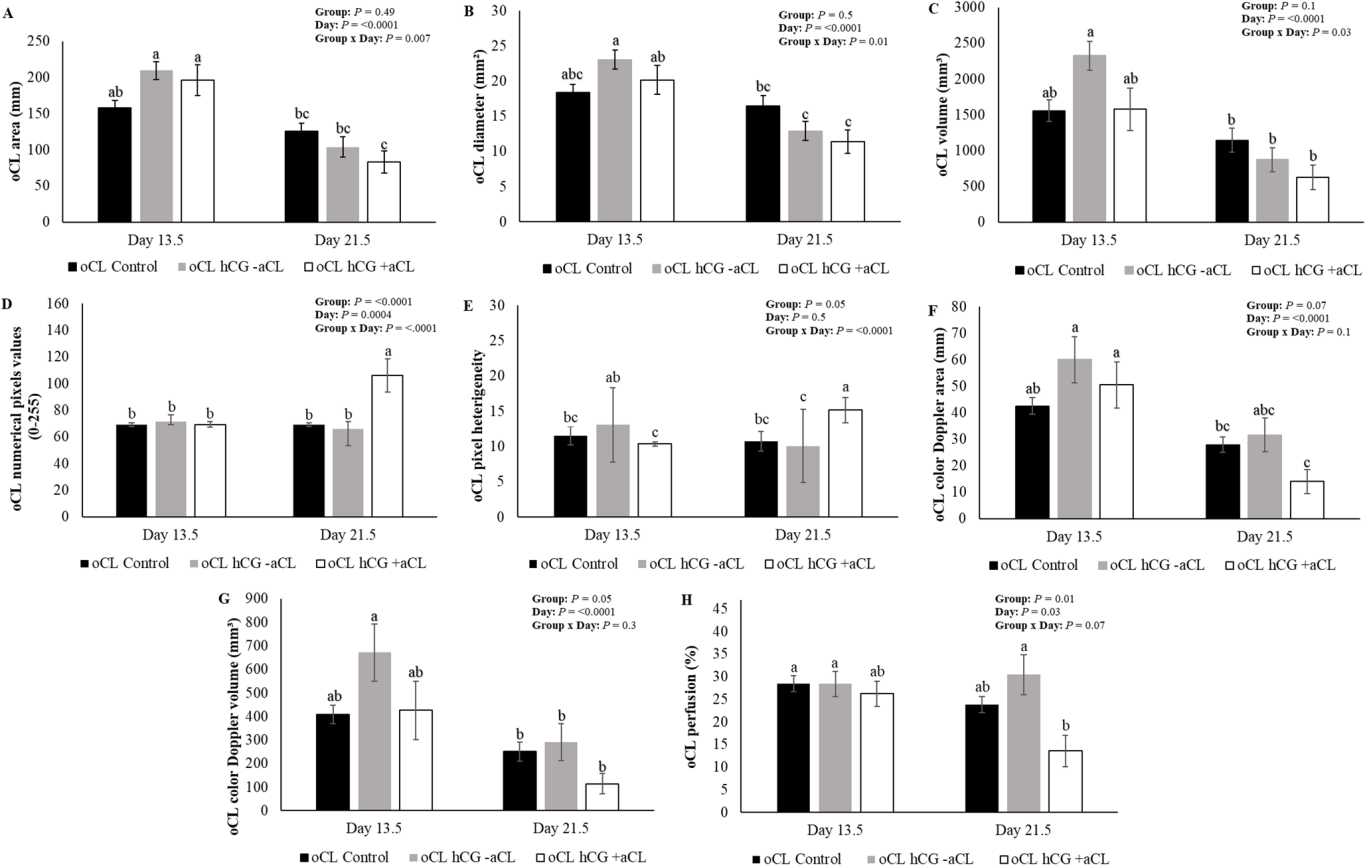
Mean (± SEM), area (**A**), diameter (**B**), volume (**C**), numerical pixels values (**D**) luteal pixel heterogeneity (**E**), color Doppler area (**F**), color Doppler volume (**G**) and perfusion (**E**) of the original corpus luteum (oCL) from G-control (oCL control), from G-hCG without the presence of aCL (oCL hCG -aCL) and from hCG with the presence of aCL in the ipsilateral or contralateral ovary (oCL hCG + aCL) on days 13.5 and 21.5 of Morada Nova ewes submitted to administration i.m. of 300 IU of hCG (G-hCG) or 1 mL of saline solution (G-Control) on day 7.5 after synchronized estrus induction treatment in the breeding season; *Different superscript letters indicate differences (*P* ≤ 0.05). *Day 13.5 - oCL Control (*n* = 30); oCL hCG -aCL (*n* = 16) and oCL hCG + aCL (*n* = 18). *Day 21.5 - oCL Control (*n* = 30); oCL hCG -aCL (*n* = 16); oCL hCG + aCL (*n* = 9)

To analyze the data presented in Figs. 2 and 3, and 4, the ewes were divided into two groups: ewes with oCL without hCG (oCL Control; *n* = 30), ewes with oCL hCG without aCL formation and ewes with oCL with the presence of aCL in the contralateral ovary (oCL hCG; *n* = 25). For the analyzes presented in Fig. 5 on day 13.5, the following were used: ewes with oCL without hCG (oCL Control; *n* = 30), ewes with oCL hCG, without aCL formation (oCL hCG -aCL; *n* = 16), ewes with oCL with the presence of aCL in the contralateral ovary and in the same ovary (oCL hCG + aCL = 18). For the analyzes presented on day 21.5, the following were used: ewes with oCL without hCG (oCL Control; *n* = 30), ewes with oCL hCG, without aCL formation (oCL hCG -aCL; *n* = 16), ewes with oCL with the presence of aCL in the contralateral ovary (oCL hCG + aCL; *n* = 9). This division was necessary due to the difficulty in distinguishing between oCL and aCL in the ipsilateral ovary across all evaluated days, with reliable differentiation only possible on Day 13.5.

## Results

There were no differences in pregnancy rates between the oCL Control (60.0%; 18/30) and the oCL hCG group (55.9%; 19/34) (*P* = 0.8). The average number of oCL per animal was 1.9 ± 0.1 in the oCL Control (56 oCL/30 animals) and 1.9 ± 0.1 in the oCL hCG group (64 oCL/34 animals) (*P* = 0.9). Within the oCL hCG group, the mean oCL without aCL formation was 2.1 ± 0.1 per animal (33/16), while the mean oCL with ipsilateral aCL formation was 1.7 ± 0.2 (15/9) and 1.9 ± 0.2 (17/9) for oCL with contralateral aCL formation. Regarding aCL formation, an average of 1.3 ± 0.2 (12/9) was observed for the ipsilateral aCL and 1.2 ± 0.1 (11/9) for the contralateral aCL.

The biometric variable values for the oCL control and oCL hCG groups on days 7.5, 10.5, 13.5 and 21.5 of pregnant and non-pregnant ewes are presented in Fig. 2. There were no interactions (*P* > 0.05) of the studied effects, treatments (G-control and G-hCG), days (7.5, 10.5 and 13.5) and PD (Pregnant and Non-Pregnant) for any of the biometric variables of the oCL (Fig. 2). The mean values of biometric variables for the oCL Control and oCL hCG groups from Days 7.5 to 13.5 are presented in Table 1. There were no differences between groups for the variables analyzed (*P* > 0.05). When comparing pregnant ewes on day 21.5 (Fig. 2), there were no differences (*P* > 0.05) for oCL area, oCL diameter and oCL volume. Similarly, no significant variations were found between Days 7.5, 10.5, and 13.5 for these biometric variables (*P* > 0.05). Dimension values of oCL on days 13.5 and 21.5 of oCL control, oCL hCG without aCL formation (oCL hCG -aCL) and oCL hCG with aCL formation present in the ipsilateral ovary or contralateral ovary (oCL + aCL) are shown in Fig. 5. Significant interactions were observed between treatments (Groups: oCL control; oCL hCG -aCL; and oCL + aCL) and days (13.5 and 21.5) (*P* < 0.05) for the variables of oCL area (Fig. 5.A), oCL diameter (Fig. 5B) and oCL volume (Fig. 5C). Regarding treatments, no differences were observed on days 13.5 and 21.5 (*P* > 0.05). Regarding the biometric variables of the contralateral aCL (*n* = 9), no differences were observed between days 10.5, 13.5, and 21.5 for aCL area (49.0 ± 10.1, 50.9 ± 8.6, and 56.45 ± 11.4; *P* = 0.8), aCL diameter (6.4 ± 1.1, 6.9 ± 0.9, and 7.5 ± 1.0; *P* = 0.7), and aCL volume (285.6 ± 91.3, 296.8 ± 71.5, and 360.5 ± 93.5; *P* = 0.8), respectively.

**Table 1:** Mean (± SEM) of luteal biometrics, color Doppler signals, and echotextural values ​​of oCL Control and oCL hCG on days 7.5, 10.5, and 13.5 of ewes treated with 300 IU of hCG 7.5 days after sponge removal

Variables	oCL Control	oCL hCG	*P*-value
oCL area (mm²)	150.9 ± 5.3	169.2 ± 7.1	0.1
oCL diameter (mm)	17.4 ± 0.6	18.3 ± 0.7	0.5
oCL volume (mm³)	1454.8 ± 78.6	1737.2 ± 102.0	0.09
oCL numerical pixels values (0-255)	70.0 ± 0.8^b^	88.8 ± 4.7^a^	0.0001
oCL pixel heterogeneity	11.5 ± 0.2^b^	14.3 ± 0.6^a^	< 0.0001
oCL color Doppler area (mm²)	48.8 ± 2.2	49.4 ± 3.1	0.8
oCL color Doppler volume (mm³)	510.0 ± 41.1	465.1 ± 20.7	0.6
oCL perfusion (%)	32.9 ± 1.0^a^	28.8 ± 1.1^b^	0.002

Different superscript letters indicate differences (*P* ≤ 0.05)

There were no interactions (*P* > 0.05) of the studied effects, treatments (oCL control and oCL hCG), days (7.5, 10.5 and 13.5) and PD (Pregnant and Non-Pregnant) for oCL numerical pixels values and oCL pixel heterogeneity (*P* > 0.05) (Fig. 3). The means of oCL Control and oCL hCG on days 7.5 to 13.5 for echotextural variables are presented in Table 1. There was a difference (*P* < 0.05) for the oCL numerical pixels values ​​and oCL pixel heterogeneity, with the oCL hCG (88.8 ± 4.7^a^ and 14.3 ± 0.6^a^) group being superior to the oCL control group (70.0 ± 0.8^b^ and 11.5 ± 0.2^b^), respectively. Differences were observed in the echotextural variables between pregnant females on day 21.5 (Fig. 3), where the oCL numerical pixels values (96.2 ± 10.2^a^ vs. 70.5 ± 1.8^b^) and oCL pixel heterogeneity (14.4 ± 1.3^a^ vs. 10.7 ± 0.3^b^) were higher for the oCL hCG group compared to the oCL control group. Regarding days 7.5, 10.5 and 13.5, there were no differences for echotextural variables (*P* > 0.05). echotextural values of oCL on days 13.5 and 21.5 of oCL control, oCL hCG -aCL and oCL + aCL there were interactions between treatments and days (*P* < 0.05) for the oCL numerical pixels values (Fig. 5D) and oCL pixel heterogeneity (Fig. 5E). In relation to treatments, the oCL numerical pixels values (Fig. 5D) showed differences (*P* < 0.0001), the oCL hCG + aCL group (81.4 ± 5.4^a^) being superior to the oCL hCG -aCL group (68.8 ± 2.6^b^) and oCL control (69.0 ± 1.0^b^). For oCL pixel heterogeneity (Fig. 5E), no differences were observed on days 13.5 and 21.5 (*P* > 0.05). Regarding the echotextural variables of the contralateral aCL (*n* = 9), no differences were observed between days 10.5, 13.5, and 21.5 for aCL numerical pixels values ​​(69.2 ± 4.1, 65.9 ± 3.3, and 58.6 ± 2.1; *P* = 0.07) and aCL heterogeneity (11.9 ± 1.6, 10.2 ± 0.5, and 9.64 ± 0.6; *P* = 0.2), respectively.

There were no interactions (*P* > 0.05) of the studied effects, treatments (oCL control and oCL hCG), days (7.5, 10.5 and 13.5) and PD (Pregnant and Non-Pregnant) for color Doppler signs oCL (Fig. 4). The means of oCL Control and oCL hCG on days 7.5 to 13.5 for the color Doppler signal variables are presented in Table 1. A difference was observed only for the oCL perfusion (*P* = 0.002), in which the oCL control (32.9 ± 1.0^a^) showed a higher percentage of perfusion compared to the oCL hCG (28.8 ± 1.1^b^). When comparing oCL control pregnant and oCL hCG pregnant on day 21.5 (Fig. 4), there were no differences (*P* > 0.05) for oCL color Doppler area, oCL color Doppler volume and oCL perfusion. When comparing the evaluation days (7.5, 10.5 and 13.5), only the oCL perfusion showed differences (*P* = 0.0002). Day 7.5 (35.1 ± 1.3^a^) was superior to day 10.5 (30.5 ± 1.2^b^) and 13.5 (28.1 ± 1.4^b^). For the color Doppler signals of oCL on days 13.5 and 21.5 of the oCL control, oCL hCG -aCL, and oCL + aCL (Fig. 4), no interactions were observed between the treatments and the evaluation days. In relation to treatments, the oCL perfusion (Fig. 5H), the oCL hCG -aCL group (29.2 ± 2.4^a^) being superior to the oCL hCG + aCL group (22.0 ± 2.5^b^) and similar to oCL control (26.2 ± 1.3^ab^) (*P* = 0.01). For the other variables, there were no differences between treatments on the same day (*P* > 0.05). For the other variables, there were no differences between treatments (*P* > 0.05). Regarding the color Doppler signal variables of the contralateral aCL (*n* = 9), no differences were observed between days 10.5, 13.5, and 21.5 for aCL color Doppler area (14.8 ± 3.3, 17.6 ± 3.9, and 14.9 ± 5.1; *P* = 0.8), aCL color Doppler volume (88.1 ± 25.5, 106.9 ± 27.6, and 100.9 ± 37.2; *P* = 0.8), and aCL perfusion (27.5 ± 4.5, 29.7 ± 4.9, and 29.9 ± 4.9; *P* = 0.8), respectively.

## Discussion

This study demonstrated that the administration of hCG on day 7.5 after estrus increased the echotextural attributes of the oCL, while biometric and color Doppler parameters remained unchanged. These findings suggest that hCG promoted functional rather than structural modifications in the luteal tissue during the early luteal phase, which is critical for maternal recognition of pregnancy (until day 13). Such echotextural changes may indicate enhanced luteinization and steroidogenic activity, which is consistent with previous reports linking echotextural attribues to luteal functionality (Vergani et al. 2020). The differentiation between the oCL and *the* aCL in our study occurred on day 13, similar to previous observations in goats (Rodrigues et al. 2022) and sheep (Fonseca et al. 2018).

The formation of an aCL, resulting from ovulation or follicular luteinization, extends luteal tissue lifespan beyond the stimulus provided by oCL stimulus. Therefore, aCL induction remains relevant in protocols where follicles ≥ 3.5 mm are present (Fernandez et al. 2019; Vergani et al. 2020). However, our results suggest that hCG had a greater effect on the oCL when an aCL was absent, indicating a potential competition for luteotropic resources or receptor availability.

A greater effect of hCG was observed on the oCL in ovaries without an aCL, likely because the luteotropic stimulation was concentrated on a single luteal structure. This response aligns with the physiological pattern of luteal development in sheep, where the maximum CL size is achieved by day 12 of the cycle (Figueira et al. 2015). The action of hCG involves binding to LH/hCG receptors, which are predominantly expressed on small luteal cells. This binding stimulates their conversion into large luteal cells that produce higher amounts of progesterone (Farin et al. 1988; Fonseca et al. 2000). Furthermore, a recent study by, (Fernández et al. 2024), observed that the administration of 300 IU of hCG in the early luteal phase in sheep led to observed greater immunodetection of steroidogenic enzymes compared to untreated animals, detecting a higher concentration of steroidogenic acute regulatory protein (StAR), which is involved in the active transport of cholesterol to the inner mitochondrial membrane, as well as an increased detection of the enzyme 3β hydroxysteroid dehydrogenase (HSD3B1), involved in the process of converting pregnenolone to progesterone. Thus, these findings reinforce the luteotropic effect of hCG. Additionally, hCG promotes fibroblast proliferation and angiogenesis by upregulating pro-angiogenic factors such as vascular endothelial growth factor (VEGF) and angiopoietin-1 (Sugino et al. 2000; Wulff et al. 2000), further supporting luteal functionality. These mechanisms explain why, even in the absence of biometric differences, echotextural improvements were observed in oCL following hCG administration. This finding is consistent with previous studies in sheep and goats (Vergani et al. 2020; Rodrigues et al. 2022).

The higher echotextural values ​​of the oCL hCG compared to the oCL control may be related to the cell differentiation promoted by the luteotropic effect of the gonadotropin. This effect, occurring either through the proliferation or the increased size of luteal cells, is fully reflected in the total pixel count, thus affecting echogenicity and heterogeneity values. Since each pixel represents the ability of a small discrete tissue unit to refract or transmit ultrasonic waves, resulting in an image displayed in various shades of gray, differences in tissue density and macromolecular composition can be indirectly analyzed by ultrasound (Duggavathi et al. 2003). This is especially relevant in studies using hormonal protocol drugs that may alter the morphometry of reproductive structures and organs in animals Duggavathi et al. 2003; Vergani et al. 2020; Rodrigues et al. 2023a).

Although hCG can act on both the oCL and the aCL, our findings indicate that its effect on the oCL may be attenuated when an aCL is present. This could be due to competition for luteotropic signals or differences in receptor distribution between the structures. Similar results were reported in goats treated with hCG, where the increase in circulating progesterone was largely associated with the additive effect of multiple CLs, rather than a significant enhancement of individual oCL function (Rodrigues et al. 2022). Therefore, while inducing aCL formation contributes to overall luteal capacity, optimizing the functional response of the oCL appears particularly relevant when aCL induction is not feasible or when the follicular reserve is limited (Fernandez et al. 2019; Vergani et al. 2020).

Despite the well-documented angiogenic potential of hCG, color Doppler evaluation revealed no significant increase in oCL hCG perfusion compared to the control group. Interestingly, perfusion was lower in the oCL of from ovaries with an aCL, suggesting a redistribution of the blood supply toward the newly formed structures. The induction of aCL formation by hCG application occurs during the period when growing antral follicles possess sufficient quantities of LH receptors. This enables aCL formation by stimulating follicular rupture and subsequent ovulation, or simply through the luteinization of large follicles (Driancourt 2001). It remains unknown whether the perfusion pattern of the aCL varies according to its origin, and whether this origin influences the hemodynamics of the oCL. Furthermore, existing studies support the hypothesis that luteal perfusion of the CL in sheep is almost maximal between the 7th and 8th day post-ovulation (Figueira et al. 2015). This physiological plateau may limit any further detectable increases in luteal blood flow following exogenous stimulation. Moreover, color Doppler ultrasonography may not fully capture microvascular remodeling or heterogeneous perfusion patterns. This limitation could explain the discrepancy observed between echotextural variations and color Doppler findings (Vergani et al. 2020; Rodrigues et al. 2022).

Echotextural analysis has been widely proposed as a non-invasive tool for assessing luteal functionality, as it reflects variations in cellular density and tissue composition (Siqueira et al. 2009; Arashiro et al. 2010; Bevilaqua et al. 2023). In the present study, hCG administration increased numerical pixels values (NPV) and pixel heterogeneity in the oCL. This suggests enhanced luteinization and collagen deposition, both of which are associated with the functional maturation of the CL. Similar associations between echogenicity and steroidogenic activity have been described in cattle and goats, where higher echotextural values correlate with increased progesterone concentrations and enhanced luteal competence (Binelli et al. 2011; Rodrigues et al. 2022). Therefore, these findings reinforce the potential of echotextural analysis as a complementary tool for monitoring luteotropic responses and predicting the functional status of the CL in small ruminants.

The luteotropic effect of hCG observed in the present study corroborates previous reports in cattle and small ruminants (Fonseca et al. 2000; Binelli et al. 2011; Rodrigues et al. 2022). Nevertheless, this impact was limited to echotextural parameters, with no significant changes in biometric or color Doppler measures. These results suggest that hCG administration promotes functional rather than structural modifications when administered on day 7.5 of the cycle. Such improvements may enhance luteal competence during the critical period of maternal recognition of pregnancy. However, it is important to emphasize that the lack of progesterone quantification and molecular markers of angiogenesis represents a limitation that warrants caution when interpreting our results. Therefore, future studies should integrate hormonal profiling, histological evaluations, and embryonic development outcomes to elucidate the physiological significance of echotextural alterations and optimize hCG-based protocols for reproductive management in sheep. These findings may support the inclusion of hCG in reproductive protocols aimed at enhancing luteal functionality, particularly when aCL formation is not expected.

## Conclusion

The administration of 300 IU of hCG on day 7.5 after sponge removal increased the echotextural attributes of the oCL in Morada Nova ewes during the breeding season, without influencing biometric or color Doppler parameters. The luteotropic effect of hCG appeared more pronounced in ovaries without aCL, suggesting that the response is modulated by the ovarian follicular context. These findings indicate that hCG may promote functional rather than structural changes in luteal tissue, supporting its potential use in reproductive protocols aimed at improving luteal competence during early pregnancy. However, further studies correlating echotextural changes with progesterone concentrations and fertility outcomes are warranted.

## Data Availability

The data that support the findings of this study are available from the corresponding author upon reasonable request.
